# Positive outcomes following Autologous Matrix-Induced Chondrogenesis (AMIC) in the treatment of retropatellar chondral lesions: a retrospective analysis of a patient registry

**DOI:** 10.1186/s12891-023-06923-8

**Published:** 2023-12-11

**Authors:** J. Gille, E. Reiss, P. Behrens, R. P. Jakob, T. Piontek

**Affiliations:** 1 Universitätsklinikum Schleswig-Holstein - Campus Lübeck, Lübeck, Germany; 2 OrthoPraxis, Zofingen, Switzerland; 3 ORTHODOK, Tonndorfer Hauptstraße 71, 22045 Hamburg, Germany; 4 Orthopaedic Department Kantonsspital Fribourg, University of Berne, Bern, Switzerland; 5 Department of Spine Disorders and Pediatric Orthopedics, University of Medical Sciences, Poznan, Poland

**Keywords:** Cartilage, Patella, AMIC, Autologous matrix-induced chondrogenesis, Chondral

## Abstract

**Background:**

The patellofemoral joint is a challenging environment for treating chondral defects. Among the surgical options for the treatment of chondral defects, the single-stage Autologous Matrix-Induced Chondrogenesis (AMIC) procedure uses a porcine collagen I/III membrane to enhance bone-marrow stimulation. However, longer term outcomes data are rare for this specific indication. In order to provide real-world information, an ongoing registry has been established to record patient data and outcomes when AMIC is used to treat chondral and osteochondral lesions.

**Methods:**

Patient data were retrieved from an ongoing, prospective, multisite registry of patients who had undergone AMIC treatment of chondral defects. We identified 64 patients who had undergone AMIC for patellofemoral chondral defects and for whom pre-operative and at least 1 post-operative score were available were included in this retrospective data analysis. Outcomes were assessed via the KOOS, VAS pain, and the Lysholm scores. Outcomes at the post-operative time-points were analysed using a factorial ANOVA with post-hoc testing while linear regression was used to assess associations between the change in the Lysholm score and lesion size.

**Results:**

There was a significant improvement in Lysholm, VAS pain, and KOOS scores from pre-operative to the 1^st^ year post-operative (*p* < 0.001), and this was maintained during the follow-up.

**Conclusions:**

The forces exerted on the patellofemoral joint make this a challenging scenario for chondral repair. Our data demonstrates that the AMIC procedure with a collagen I/III membrane is an effective treatment for retropatellar cartilage lesions, and provides reliable results, with decreased pain and improved function. Importantly, these improvements were maintained through the follow-up period.

## Introduction

Articular cartilage is essential to the proper functioning of synovial joints, as it transmits loads and provides an articulating surface with a low coefficient of friction [1, 2]. Unfortunately, articular cartilage has minimal regenerative capacity due to its avascular and hypocellular character [3]. In the patellofemoral joint (PFJ), this low friction environment is essential as the patella glides in the trochlear groove, enhancing the moment arm of the quadriceps [4] as well as contributing to the optimization of control strategies in locomotion [5]. While patellofemoral compressive forces have been calculated to be somewhat more than 4 times body weight [6] there is also data that shows these can be as high as 11 times body weight [7]. Thus, the gliding movement and the high compressive forces create a challenging environment for articular cartilage.

In considering the biomechanical complexity and the forces exerted on the articular surfaces, it is not surprising that the patellofemoral (PF) joint is a common site of pathology, whether chronic or acute. A systematic review had noted that PF defects comprised 37% of the knee chondral lesion sites [8]. This is consistent with the conditions that had been recorded for over 5000 arthroscopic surgeries, in which it was reported that PF defects accounted for the majority of the lesions [9].

While first-line treatment will often be conservative management [10], persistent symptoms may indicate a need for surgery [11]. Among surgical options, a single-stage procedure known as autologous matrix induced chondrogenesis (AMIC) uses bone marrow stimulation techniques and then covers the treated site with a bilayer collagen I/III membrane (Chondro-Gide®, Geistlich Pharma AG, Wolhusen, Switzerland) in order to protect the blood clot that contains the factors released from the bone marrow stimulation, thus providing a biological chamber for repair tissue to mature [12, 13]. However, the data that has focused specifically on patellar lesions has been limited, with relatively short follow-up [14] or small cohorts [15, 16]. With a paucity of data on the repair of PF lesions, we conducted a retrospective review of patient outcomes, using data that had already been entered into a registry, in order to assess the role that this procedure can play in treating focal chondral defects on the retropatellar surface.

## Methods

### Study design

This study is an analysis of the data of patients who had been treated with AMIC and were prospectively enrolled in a registry. The registry is an ongoing, multicentre database designed to longitudinally track changes in function and symptoms in patients who have undergone repair of chondral lesions via this procedure [17, 18]. Documentation was made on electronic case report forms, with surgeons having access to the registry via a web interface. Surgeons had access to their own patients’ data, whereas the summary and overall performance data were anonymized. All patients were educated and informed in detail about the AMIC technique as well as alternative procedures. Thereafter, the patients who chose to undergo AMIC were enrolled in the registry. All patients signed an informed consent to participate in the registry, and all treatment and follow-up examinations followed the standard of care, with no additional visits imposed on the patients. Ethical approval for this study was obtained from the ethics review board of the University of Lübeck (file no. 19–178). Because the registry has no provision for radiographic follow-up, data regarding the development of radiographically verified osteoarthritis were not available.

### Patients

The data was based on a prospective registry for AMIC patients, which has previously been published concerning a larger cohort [18]. Patients were included in this analysis if their retropatellar chondral lesion had been treated via AMIC and they had completed pre- and post-operative measures of knee function and pain. The indication for chondral repair was a symptomatic, circumscribed cartilage lesion on the retropatellar surface, with an Outerbridge classification of grade III or IV. The main exclusion criteria were concomitant surgery at the time of the index procedure (e.g. ACL reconstruction), advanced osteoarthritis, significant narrowing of the joint lines, underlying rheumatic disease, total meniscectomy, or deviation of the mechanical axis of the affected compartment. Baseline data collection included surgical history, lesion size, concurrent procedures, age, BMI, and sex. Data were collected at baseline and at each subsequent folow-up visit, which had followed the standard of care. As this was a registry-based study, including all patients who fulfilled the inclusion and exclusion criteria, we did not perform an a priori power analysis.

### Treatment

The operative procedure was performed through either a mini-open approach or arthroscopically, at the discretion of the surgeon.

**Fig. 1:**
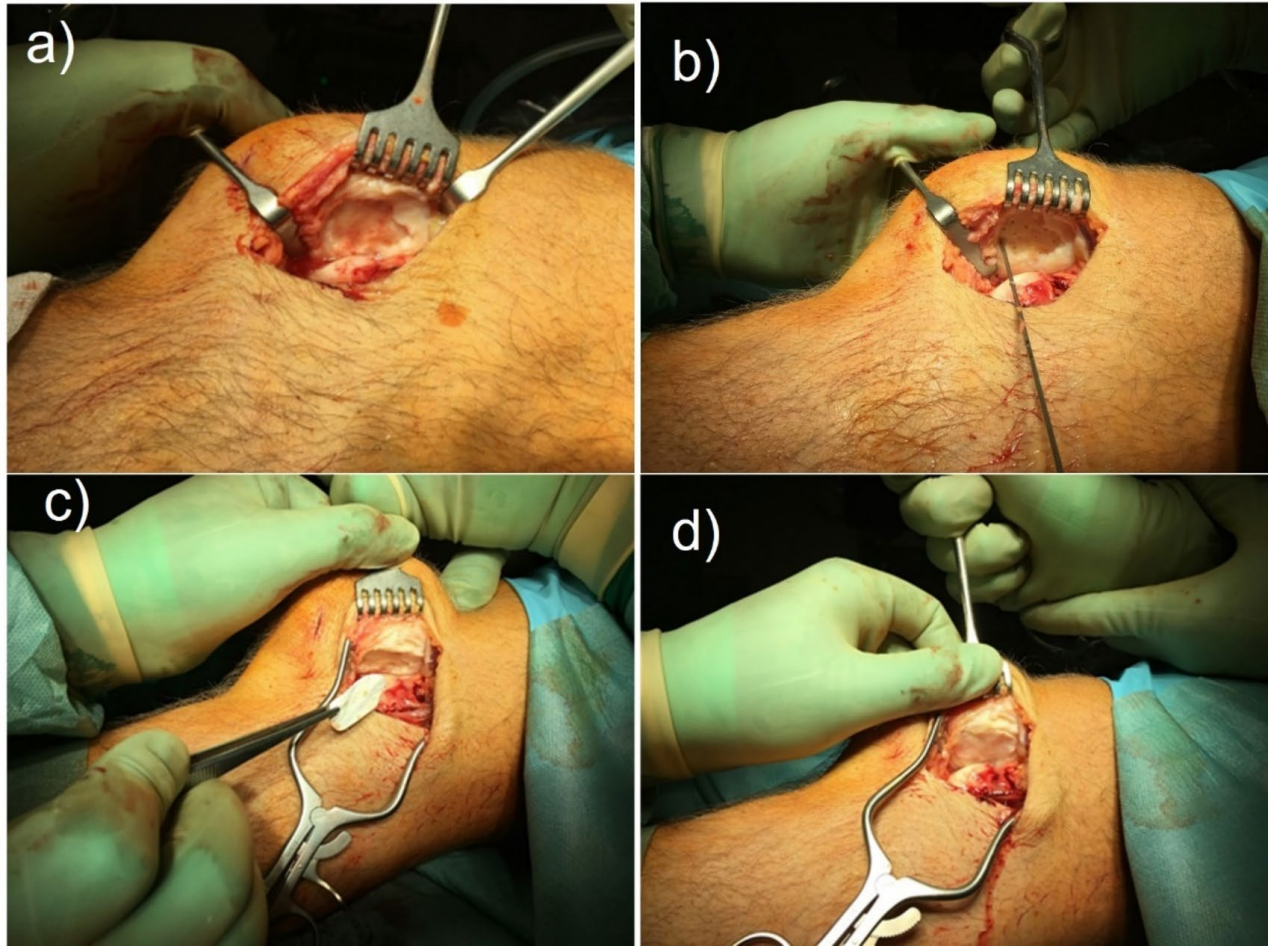
**a** Prepared retropatellar defect with a stable, perpendicular cartilage shoulder surrounding the defect; **b** bone marrow stimulation using a k-wire; **c** inserting cut membrane into prepared defect; **d** Chondro-Gide membrane in place

After debridement and careful removal of degenerative and loose cartilage fragments bone marrow stimulation was performed using a 1.2mm drill or k-wire in order to perforate the subchondral bone plate, typically to a depth of approximately 1 cm, resulting in bleeding and release of bone marrow stem cells and other factors into the defect. The prepared chondral defect was then covered with a collagen I/III membrane of porcine origin (Chondro-Gide®, Geistlich Pharma AG, Wolhusen, Switzerland) that was previously cut to fit the defect size by using an aluminium template. Next, the membrane was fixed either by sutures or a fibrin sealant, depending on the surgeon’s preference. Previous research has shown that these 2 fixation methods provide equivalent results. [19, 20]. The knee joint was held in an extended position for 5 min before the joint was flexed ten times to test the stability and position of the matrix. Figure 1 illustrates the steps during an open AMIC procedure. The incision was then closed in layers with standard techniques, and a drainage without suction was applied. The knee was immobilized for the first few days followed by continuous passive motion with restricted knee angles and limited weight bearing for approx. 6 weeks.

### Outcomes assessment

At each follow-up, patients rated their pain using the VAS, with 0 indicating no pain and 10 indicating the worst pain the patient has known, while functional outcomes were assessed using the Lysholm score and the KOOS, both of which are validated, functional scores. [21, 22]. Because the data from this study were based on a registry, which followed standard of care, there were no additional, predefined, clinical follow-up visits. Each clinic had maintained contact with their patients, and actively motivated them to adhere to the follow-up protocol as well as sending follow-up questionnaires to patients. There was no radiographic follow-up after AMIC in the registry, and additional radiographic exams had not been described in the original consent and were not part of the standard of care follow-up. Consequently, data regarding the development of radiographically verified osteoarthritis are not available. Patients were not financially compensated for their time in completing the data-collection forms.

### Statistical analysis

We did not conduct a power analysis to estimate the sample size, as this registry study included all patients who had undergone AMIC surgery for the repair of a patellar chondral lesion. The outcome variables, VAS pain, KOOS subscales and Lysholm, were evaluated via a factorial analysis of variance (ANOVA) across all time points. Exploratory analyses were conducted to test for relationships between the change in scores from baseline and the patients’ BMI, age and defect size. The a priori alpha level was set at *p* = 0.05. Statistical analyses were performed using MedCalc, version 19.4 (MedCalc Software, Ostend, Belgium).

## Results

The current registry database was queried in order to find all patients who had been treated for a patellar, chondral lesion by one of the 5 co-authors. Of the 86 patients that were identified, only those who had pre-operative and at least 1 post-operative outcome measure were included in the statistical analysis. There were no other exclusion criteria. This resulted in 64 patients being included in this report. The number of patients at each time point is shown in Table 1.

**Table 1:** The number of patients with available data at each time point

Visit	n
Pre-operative	64
Year 1	40
Year 2	24
Year 3	17
Year 4	12
Year 5	7
Year 6	11
Year 7	4
Year 8	6
Year 9	11
Year 10	10

### Patient demographics

The most common surgical approach was mini-open, the most common fixation method was using fibrin glue and the mean length of follow-up was 5.8 years with a range from 1 – 10 years. Of the 64 patients included in this analysis, 56 patients have a follow-up of 2 or more years. The patient demographics are presented in Table 2. There were no significant differences when the lesion size, age or BMI were compared relative to the sex of the patient. Of these patients, sport was the most commonly listed cause of the injury.

**Table 2:** The characteristics of the patients that were included in this study

	N	Lesion size (cm^2^)	Age (years)	BMI
Male	32	2.9 ± 1.4	36.1 ± 15.4	24.9 ± 3.2
Female	33	3.1 ± 1.4	33.7 ± 11.6	23.9 ± 5.2

### Lysholm

**Fig. 2:**
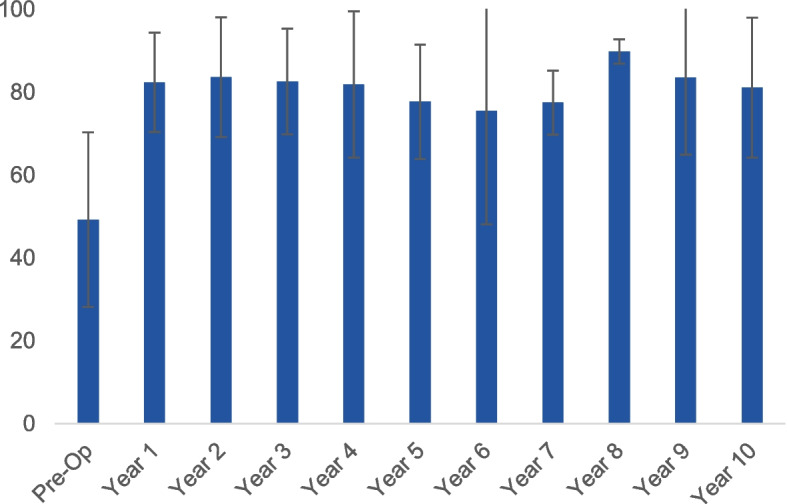
The scores for the Lysholm test across all time points

There was significant difference in the scores (*P* < 0.001) and post-hoc tests revealed that the pre-operative Lysholm score was significantly different from all post-operative scores, as depicted in Fig. 2. There were no significant differences noted between any of the post-operative scores.

### KOOS Scores

**Fig. 3:**
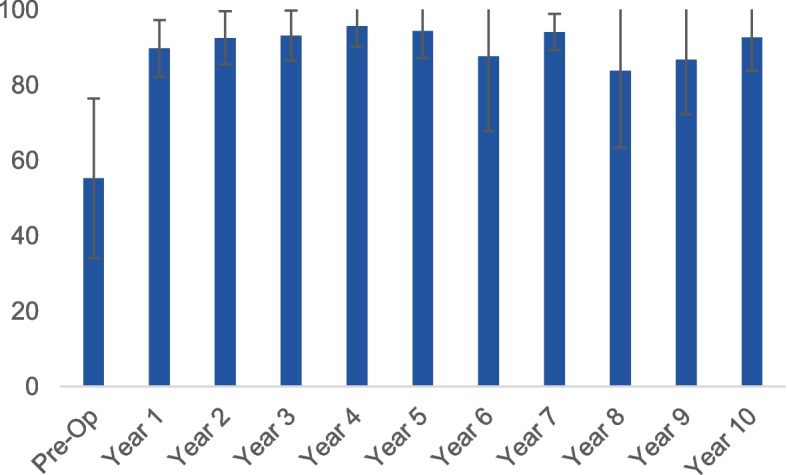
The scores for the KOOS subscale for symptoms, at each time point in the follow-up period

**Fig. 4:**
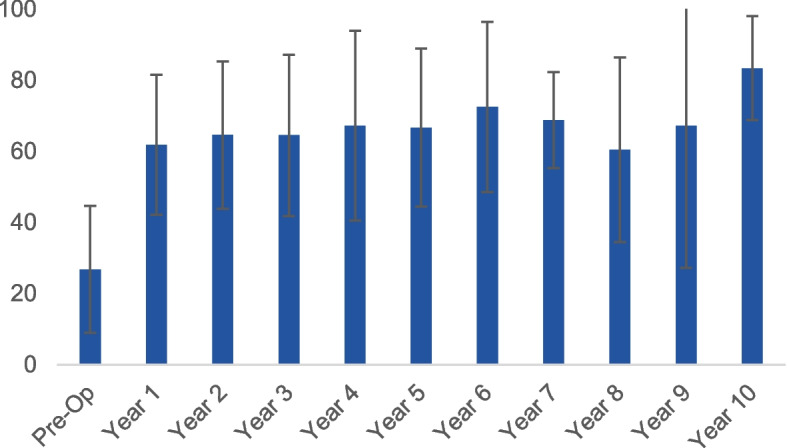
QoL subscale over the follow-up period

According to recommendations for use of the KOOS score, [23] we did not take into account the total KOOS in our results but separately evaluated the 5 domains (symptoms, pain, quality of life, sport and leisure and activities of daily life). We noted a significant improvement in all the domains of the KOOS. The KOOS score for symptoms improved from a mean of 48.7 ± 23 to a mean of 87.5 ± 12.5 (*p* < 0.001) (Fig. 3) The pain subscale improved from a mean of 52.4 ± 16.3 to 89.8 ± 13.2 (*p* < 0.001), while quality of life (QoL) more than doubled, going from 26.8 ± 178 pre-op to 83.3 ± 14.6 post-operatively (*p* < 0.001) (Fig. 4) and sports and leisure activities showed a similarly large improvement, rising from 21.7 ± 14.4 pre-operatively to 85 ± 12.6 post-operatively (*p* < 0.001) while the KOOS domain for activities of daily living (ADL) improved from 55.2 ± 21.2 pre-operatively to 92.6 ± 8.8 post-operatively (*p* < 0.001).

### Pain

**Fig. 5:**
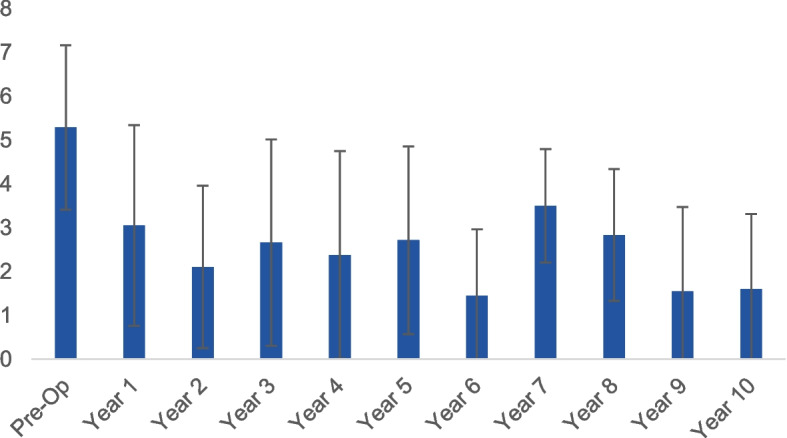
The change in VAS over time

Similar to the previous outcomes that have been listed, the VAS score for pain (Fig. 5) showed a significant difference between the pre-operative mean of 5.7 ± 1.8 and all post-operative scores (*p* < 0.001). It should be noted that there were no significant differences between the pain score at any of the post-operative time points.

### Regressions

**Fig. 6:**
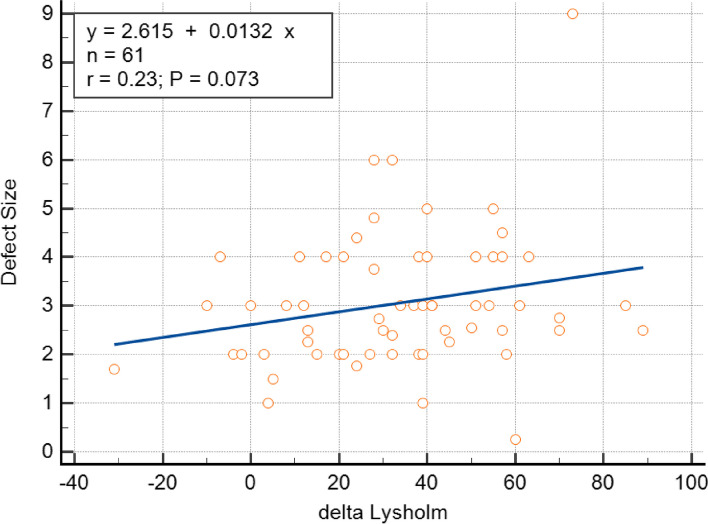
The regression plot that shows the change in Lysholm score relative to the patient's defect size

The exploratory analysis examined relationships between demographic variables and the Lysholm score at the last follow-up. While neither age nor BMI showed a relationship with the Lysholm score at the last follow-up, there was a trend (*p* = 0.073) towards a relation between defect size and improvement in Lysholm score, as shown in Fig. 6. Although not significant, the data suggests that patients with larger defects tended to exhibit greater changes in Lysholm score at their last follow-up visit.

### Responder rate and MCID

In evaluating clinical data, we also considered the minimal clinically important difference (MCID) in the outcomes. This has been reported to be 10.1 points for Lysholm [24] while the KOOS Sports and Recreation subscale has been reported to be 30 points, [25]. Therefore, among the patients who exceeded the MCID for Lysholm at their last follow-up visit, we noted a responder rate of 0.83 for the Lysholm tests. For the sport and recreation subscale of the KOOS, the responder rate was 0.84.

### Complications and failures

When the registry was initiated in 2002 it was not designed to capture complications and failures. Although a review of the patient records at each site did not reveal complications in this cohort, the loss to follow-up may have been influenced by reoperations or failures.

## Discussion

The results of this study, showing a sustained improvement in patient outcomes for several years after surgery, supports the use of AMIC Chondro-Gide® as a surgical treatment of chondral lesions of the patella. We observed a significant improvement between the pre-operative and post-operative scores related to function (Lysholm and KOOS) as well as a significant reduction in pain (VAS). Among our patients, the improvements in all KOOS subscales, well above the MCID and with a correspondingly high responder rate, indicates that the treatment is an effective means of treating patellar chondral lesions. Since there were no significant differences noted between any of the post-operative time points it seems apparent that the improvements in the patients’ outcomes are maintained throughout the follow-up period of this study.

These results are consistent with our own experience in the knee, in which we noted positive outcomes up to 7 years postoperatively [18]. In that dataset, we had recorded outcomes after treatment of lesions on all articulating surfaces of the knee. The results from our earlier paper and our current patellofemoral data are consistent with studies that have reported positive longer-term outcomes in the hip [26] and in the ankle [27] when AMIC with Chondro-Gide was used in the repair of focal chondral lesions.

In addition to the results we have reported here, as well as our previous data [18] other publications have detailed positive patient outcomes following AMIC in the knee. A case series of patients treated with AMIC for large chondral knee defects has shown consistent improvements in patient outcomes over 7 years [28]. An RCT, which investigated the use of biological adjuvants in the AMIC procedure, had reported sustained benefits up to 9 years status post [29]. Specific to the patellofemoral joint, a small cohort study with 5 years follow-up had stated that AMIC is considered to be a reliable one-step alternative for the treatment of large, isolated patellar cartilage defects [16].

While MFx is often considered a first line treatment for focal, chondral defects, due to its simplicity and low-cost, the data on its durability, in terms of pain relief and improved function, may make other options more attractive. A randomized controlled trial (RCT) that compared microfracture (MFx) to AMIC reported that while AMIC showed a sustained benefit, the outcomes for MFx patients started to worsen between the 2-year and 5-year follow-ups [19]. Similarly, it was seen in a case–control study with 4-year follow-up that the AMIC procedure for the treatment of patellar chondral defects results in better IKDC and Lysholm scores along with a significant reduction of the VAS score [30]. Additionally, the Tegner scale demonstrated that patients exhibited a higher level of activity after AMIC, relative to MFx, while the AMIC group evidenced a lower rate of failure [30].

Additionally, we took into consideration the MCID in the assessment of outcomes. The MCID for Lysholm and VAS has been reported to be 10.1 and 2.7, respectively [24] and among this cohort, we had a responder rate of 0.83, which is somewhat better than the responder rate of 0.7 that was recently reported for ACI [31]. Considering that a majority of patients presented with injuries resulting from sports activities, we also felt it would be worthwhile to examine the responder rate with regard to the KOOS sport and recreation subscale, with an MCID calculated as 30 points [25]. Our patients cohort showed a responder rate of 0.84 for this subscale.

Recent data has noted that concomitant corrective surgery for patellar instability results in low failure rate with satisfactory clinical outcomes at mid-term follow-up [14]. Among the cohort in our study, corrective tibial osteotomies for some patients had been recorded, but the numbers do not allow for a meaningful comparison of outcomes data with regard to this aspect of the procedure. The necessity of physiological knee function, as a component of chondral repair, was clearly emphasized with regard to proper alignment being a critical factor in long-term success of cartilage repair [32]. Indeed, it has recently been stated that cartilage repair without respecting alignment is fruitless [33].

Certainly, our study is not without limitations. While a registry collects real-world data based on all patients, the follow-up can be problematic. In contrast to an RCT with dedicated time points, the follow-up in a registry simply follows standard of care. As an example, if a patient is unsatisfied with their treatment and they seek treatment elsewhere, then they will be lost to follow-up, thus reinterventions or revision surgery will not be captured. In the recent study the number of patients who had data at each post-operative time point decreased as time progressed from the baseline evaluation, which is a limitation of the registry and one that would have certainly decreased the power of the statistical analysis. With regard to the statistical analysis, in a clinical study in which the patients are seen at several pre-established timepoints, a repeated-measures analysis of variance is typical. However, this registry had collected post-operative data on patients when they were seen in routine visits that followed standard of care. Thus the lack of planned visits, set to a time schedule in the study protocol, precluded a repeated-measures ANOVA. Therefore, the factorial analysis, which may limit the power of the study, was a realistic method of statistical analysis. Furthermore, we did not collect radiographs outside of the normal standard of care. While an assessment of such images can provide research utility, all of the surgeons would have used imaging as part of the normal treatment and would have detected any anomalies in the course of treatment. For these reasons, as well as ethics and cost, there were no additional radiographs.

## Conclusion

The complexity and mechanical demands of the patellofemoral joint create a challenging environment for the repair of focal chondral defects. The results of our ongoing registry study, in which patients reported significant, sustained improvements in KOOS, Lysholm and pain, support the use of the AMIC procedure as an effective treatment for the repair of chondral lesions on the retropatellar surface.

## Data Availability

Datasets generated and analysed during current study are not publicly available due to controlled personal data agreement and data security.
